# Age-Dependent TLR3 Expression of the Intestinal Epithelium Contributes to Rotavirus Susceptibility

**DOI:** 10.1371/journal.ppat.1002670

**Published:** 2012-05-03

**Authors:** Johanna Pott, Silvia Stockinger, Natalia Torow, Anna Smoczek, Cornelia Lindner, Gerald McInerney, Fredrik Bäckhed, Ulrich Baumann, Oliver Pabst, André Bleich, Mathias W. Hornef

**Affiliations:** 1 Institute for Medical Microbiology and Hospital Epidemiology, Hannover Medical School, Hannover, Germany; 2 Institute of Animal Breeding and Genetics, Veterinary University of Vienna, Vienna, Austria; 3 Laboratory for Animal Science, Hannover Medical School, Hannover, Germany; 4 Institute of Immunology, Hannover Medical School, Hannover, Germany; 5 Department of Microbiology, Tumor and Cell Biology, Karolinska Institutet, Stockholm, Sweden; 6 Sahlgrenska Center for Cardiovascular and Metabolic Research/Wallenberg Laboratory, University of Gothenburg, Gothenburg, Sweden; 7 Clinic for Paediatric, Kidney-, Liver and Metabolic Diseases, Hannover Medical School, Hannover, Germany; Fox Chase Cancer Center, United States of America

## Abstract

Rotavirus is a major cause of diarrhea worldwide and exhibits a pronounced small intestinal epithelial cell (IEC) tropism. Both human infants and neonatal mice are highly susceptible, whereas adult individuals remain asymptomatic and shed only low numbers of viral particles. Here we investigated age-dependent mechanisms of the intestinal epithelial innate immune response to rotavirus infection in an oral mouse infection model. Expression of the innate immune receptor for viral dsRNA, Toll-like receptor (Tlr) 3 was low in the epithelium of suckling mice but strongly increased during the postnatal period inversely correlating with rotavirus susceptibility, viral shedding and histological damage. Adult mice deficient in Tlr3 (Tlr3^−/−^) or the adaptor molecule Trif (Trif*^Lps2/Lps2^*) exerted significantly higher viral shedding and decreased epithelial expression of proinflammatory and antiviral genes as compared to wild-type animals. In contrast, neonatal mice deficient in Tlr3 or Trif did not display impaired cell stimulation or enhanced rotavirus susceptibility. Using chimeric mice, a major contribution of the non-hematopoietic cell compartment in the Trif-mediated antiviral host response was detected in adult animals. Finally, a significant age-dependent increase of *TLR3* expression was also detected in human small intestinal biopsies. Thus, upregulation of epithelial TLR3 expression during infancy might contribute to the age-dependent susceptibility to rotavirus infection.

## Author Summary

Intestinal epithelial cells line the mucosal surface of the gut. They are therefore the first to encounter orally ingested pathogenic microorganisms such as rotavirus, a frequent cause of diarrhea both in humans and other mammals. Recognition of structural components of microbial pathogens is facilitated by specialized immune receptors that allow cells to mount an early host defense and restrict infection. Since rotavirus-induced disease is largely restricted to human infants and mice during the postnatal period we studied the age-dependent epithelial expression of immune receptors involved in rotavirus recognition. Strikingly, expression of one such immune receptor, Toll-like receptor 3 (Tlr3), precisely correlated with the establishment of resistance against rotavirus infection in mice. Using an oral rotavirus infection model, we confirmed that Tlr3-induced immune responses contributed to restrict rotavirus replication in adult but not neonate animals. Expression of the same innate immune receptor was also increased with age in human gut biopsies. We thus conclude that the low intestinal epithelial Tlr3 expression contributes to the age-dependent susceptibility towards rotavirus infection.

## Introduction

Rotavirus is one of the most common causes for infectious gastroenteritis in human children and contributes to the high infant mortality in countries with limited access to medical care [1]. It particularly affects infants younger than six years, whereas adults do not develop symptomatic disease. Similar to the situation in humans, neonate mice are highly susceptible to oral rotavirus infection and develop symptomatic disease. Rotavirus exhibits a marked cell tropism for epithelial cells of the small intestine and causes significant destruction of the neonatal epithelium. Histological changes are host specific and include epithelial vacuolization and villous blunting. During the third week of life, however, the susceptibility of mice to infection decreases markedly with reduced intestinal viral replication and no signs of clinical disease observed in adult animals [2]. Since the resistance of adult mice to rotavirus infection is also acquired under rotavirus-free breeding conditions, the age-dependent susceptibility appears not to be mediated by an adaptive cellular or humoral immune response. Developmental aspects of the intestinal mucosa or the immaturity of the enteric innate immune system might therefore account for the particular susceptibility of neonate individuals.

Activation of the innate immune system is based on the recognition of conserved microbial structures by a limited number of membrane and cytosol resident receptor molecules. Viral nucleic acid molecules are recognized by the endosomal Toll-like receptors (Tlrs) 3, 7, 8, and 9, cytosolic retinoic acid-inducible gene-like receptors (RLRs) Rig-I and Mda5, and the dsRNA-dependent protein kinase R (Pkr) [3]. Tlr3 and the RLRs Rig-I and Mda-5 recognize double stranded RNA (dsRNA) molecules, whereas Tlr7/8 and Tlr9 sense single stranded RNA (ssRNA) and DNA, respectively. The endosomal Tlrs 7, 8 and 9 signal via the adaptor protein myeloid differentiation primary response gene 88 (MyD88), whereas Tlr3 uses the TIR domain containing adaptor inducing interferon-β (Trif) adaptor molecule. RLRs signal via the common adaptor mitochondrial antiviral signaling protein (MAVS). Ligand-mediated activation of both, the Tlr pathway as well as the RLR pathway, leads to activation of NF-κB, MAP kinases and IRF3 and thereby to the induction of proinflammatory and antiviral response genes [4].

The best-characterized rotavirus-derived molecular pattern recognized by the innate immune system is the dsRNA genome and its replication intermediates. In accordance, *in vitro* data demonstrated the involvement of Tlr3 in the innate immune recognition of reo- and rotavirus RNA and intact rotavirus particles [5]–[7]. Additionally Rig-I, Mda-5 and Pkr have been reported to contribute to the immune response to rotavirus infection *in vitro* and *in vivo* [8]–[10]. In the present study, we investigated whether changes in the intestinal epithelial expression level of innate immune receptor molecules during postnatal development might contribute to the age-dependent susceptibility to rotavirus infection. We found that *Tlr3* but not *Tlr7*, *Pkr*, *Rig-I* or *Mda-5* was highly upregulated in murine intestinal epithelium during the postnatal period. Age-dependent *TLR3* upregulation was also found in human small intestinal biopsies. In addition, Tlr3-Trif mediated innate immune signaling significantly influenced epithelial cytokine and chemokine expression, antiviral effector cell recruitment and viral shedding in adult but not neonate mice. Our results identify the postnatal increase in the intestinal epithelial expression of Tlr3 as a key factor determining the age-dependent susceptibility to rotavirus infection.

## Results

### Age-dependent pattern recognition receptor (PRR) expression, ligand responsiveness and rotavirus susceptibility

**Figure 1:**
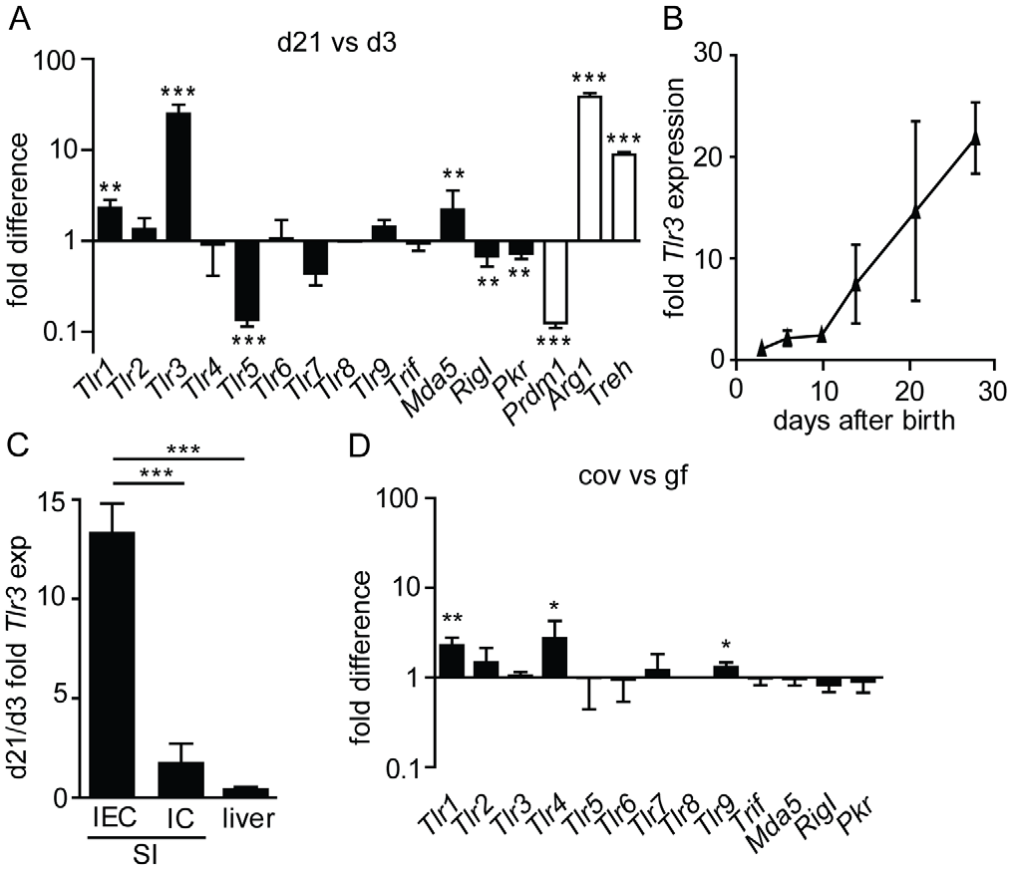
Age-dependent epithelial PRR expression. (**A and D**) Expression levels of certain innate immune receptors as indicated in the figure were comparatively analysed by microarray analysis in IECs isolated from 3-day-old *versus* 21-day-old mice (**A**) and from 28-day-old germ-free *versus* conventional mice (**D**). (**B**) IECs of 2, 4, 9, 14, 20 and 27-day-old mice were analyzed by quantitative RT-PCR for the expression level of *Tlr3* (n = 3/time point). The values were normalized to the expression of *Gapdh*. (**C**) *Tlr3* expression levels in liver tissue (n = 3), isolated small intestinal IECs (n = 3) and immune cells (IC, 3-day-old mice: n = 10, 21-day-old mice: n = 12) were determined by quantitative RT-PCR and normalized to the expression of *Gapdh*. Data represent means ± SD (*p<0.05; **p<0.01; ***p<0.001, unpaired t test).

Significant changes in the gene expression profile of intestinal epithelial cells (IEC) are found during the postnatal period [11], [12]. To investigate a possible influence of age-dependent epithelial gene expression on infection susceptibility, the expression level of Tlrs, the helicases RigI and Mda5 and Pkr was compared in IECs isolated from 3-day-old suckling mice and weaned 21-day-old mice. Microarray analysis demonstrated that the expression level of *Tlr3* is dramatically increased in adult compared to neonatal epithelium (Figure 1A). *Tlr3* upregulation during the postnatal period reached similar levels as other established age-dependently upregulated murine genes such as *Arginase 1* (*Arg1*) and *Trehalase* (*Treh*). In contrast, expression of *Tlr2*, *4*, *6*, *7*, *8*, and *9* as well as the Tlr3 adaptor molecule *Trif* remained unchanged. A low but significant upregulation of *Tlr1* and *Mda-5* and down-regulation of *Tlr5, RigI*, and *Pkr* were noted. Also, expression of the recently established epithelial transcriptional repressor *PR domain containing protein 1* (*Prdm1*) also called *B lymphocyte-induced maturation protein 1* (*Blimp1*) was significantly downregulated during the postnatal period, as expected [11], [12]. The enhanced epithelial expression of *Tlr3* was subsequently confirmed by quantitative RT-PCR (Figure 1B). Whereas *Tlr3* expression remained low during the first 10 days after birth, a steady increase was observed between day 10 and day 21 after birth leading to a 20-fold increase in IECs of 28-day-old mice compared to newborn animals. In contrast to the highly significant upregulation in IECs, *Tlr3* expression in isolated intestinal immune cells or liver tissue remained largely unchanged during the same period (Figure 1C). To investigate a possible influence of the enteric microbiota established during the neonatal period, IECs isolated from 28-day-old conventional and germ-free bred animals were analyzed. No difference in the expression level of TLR3 was noted indicating that no microbial stimulus is responsible for the increased expression of *Tlr3* during the postnatal period (Figure 1D). Similarly, expression of *Tlr2, 5, 6, 7, 8, Mda5, RigI*, *Pkr* and *Trif* remained unchanged. A moderately lower expression in the absence of the enteric microbiota was observed for *Tlr1, Tlr4* and *Tlr9*.

**Figure 2:**
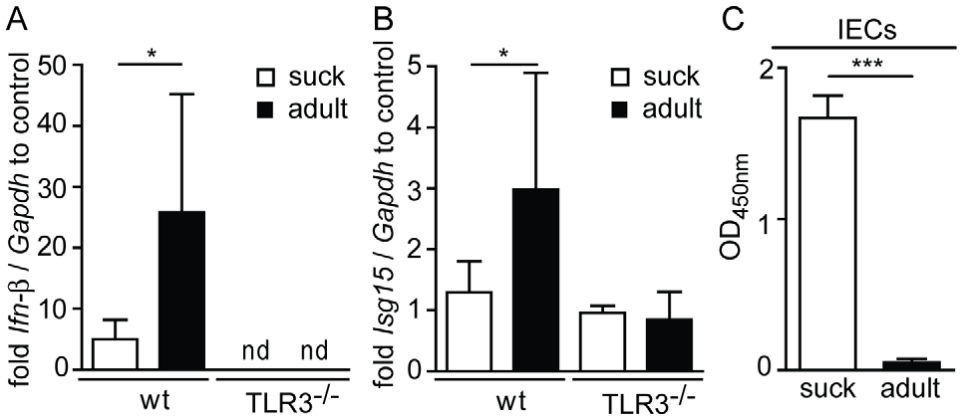
Age-dependent epithelial Tlr3 responsiveness and susceptibility towards rotavirus infection. (**A and B**) Gene expression in IECs from suckling (3–7-day-old) and adult (21–28-day-old) mice following intraperitoneal injection of 50 ng poly(I∶C)/g body weight was examined. *Ifn-β* (**A**) and *Isg15* (**B**) expression in isolated IECs 2 h after injection was analyzed by quantitative RT-PCR. (**C**) Viral load in IECs of suckling (n = 6) *versus* adult (n = 4) mice at day 4 after oral infection with the murine rotavirus strain EDIM was determined by ELISA. Data represent means ± SD (*p<0.05; ***p<0.001, unpaired t test).

Next, we examined the functional consequences of low *Tlr3* expression in the neonatal intestinal epithelium *in vivo* using the established Tlr3 ligand poly(I∶C). A body weight adjusted low dose of poly(I∶C) was administered intraperitoneally to suckling and adult mice and the expression of established Tlr3-induced genes was analyzed in isolated IECs. No increase of *Ifn-β* and *Isg15* expression was observed in IECs isolated from suckling wildtype mice or adult *Tlr3^−/−^* animals. In contrast, *Ifn-β* and *Isg15* mRNA levels were significantly induced by poly(I∶C) in IECs from adult wild-type mice, indicating a functionally relevant upregulation of intestinal epithelial Tlr3 expression during the postnatal period (Figure 2 A and B).

Also, we compared the age-dependent *Tlr3* expression to rotavirus susceptibility using a murine oral infection model. Suckling mice at day 5 after birth and 28-day-old adult animals were orally infected with the murine rotavirus strain EDIM. Suckling mice developed diarrhea approximately at day 2 post infection (p.i.), which lasted for up to 5 days, whereas no clinical sign of infection (i.e. diarrhea) was observed in adult animals in accordance with previous reports on the age-dependent susceptibility to rotavirus (data not shown) [2]. Rotavirus replicated much more efficiently in the epithelium of suckling as compared to adult mice, as demonstrated by the markedly enhanced viral load in isolated IECs from neonatal animals (Figure 2 C). Finally, major histopathological changes such as epithelial vacuolization and tissue disruption were noted in small intestinal tissue section of virus-infected suckling mice (Figure S1 A). In contrast, intestinal tissue from infected adult animals was indistinguishable from tissue sections obtained from healthy uninfected control animals (Figure S1 B). Together, these data confirm the age-dependent susceptibility towards rotavirus infection and show that rotavirus susceptibility correlates with low expression of Tlr3 and responsiveness in intestinal epithelial cells.

### Tlr3 and Trif contribute to antiviral protection in adult but not neonatal mice

**Figure 3:**
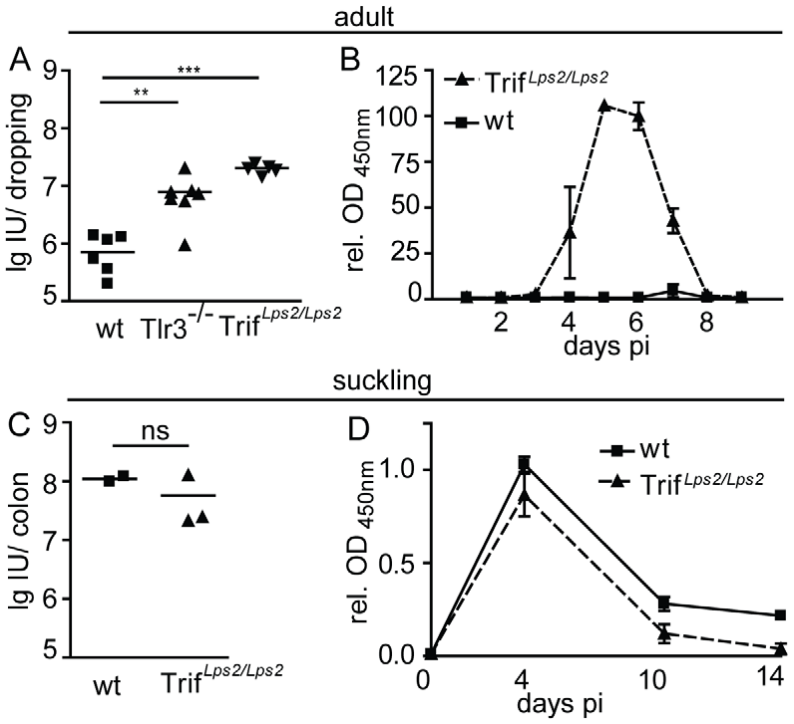
Rotavirus susceptibility of Trif*^Lps2/Lps2^* and Tlr3^−/−^ mice. (**A**) Adult wild-type (wt) (n = 6), Tlr3^−/−^ (n = 7) and Trif*^Lps2/Lps2^* (n = 5) mice were orally infected with rotavirus EDIM and shedding in fecal droppings was monitored by ELISA at day 4 p.i. in parallel with a serial dilution of a RRV virus stock with known virus titer to facilitate determination of infectious units (IU). (**B**) Adult wild-type (wt) and Trif*^Lps2/Lps2^* mice were orally infected with rotavirus EDIM and monitored for rotavirus antigen shedding between day 1 and day 10 p.i. (wt n = 8; Trif*^Lps2/Lps2^* n = 7). The relative OD_450 nm_ values normalized to the values obtained from wt animals at day 4 p.i. are shown to facilitate comparison of independent experiments. (**C**) Suckling wt and Trif*^Lps2/Lps2^* mice were orally infected with rotavirus EDIM and colon homogenates were analyzed for IU at day 4 p.i. as described for (A). (**D**) OD_450 nm_ values normalized to the values obtained from wt animals at day 5 p.i. were measured in colon homogenates of suckling wt and Trif*^Lps2/Lps2^* mice at day 5, 10 and 14 p.i. (n = 3, time point and genotype). (*p<0.05; **p<0.01, ***p<0.001, Mann-Whitney test).

Next, we investigated the role of Tlr3- and Trif-mediated signaling for rotavirus recognition and antiviral host defense in the context of both neonatal and adult rotavirus infection. Adult wild-type, Tlr3^−/−^ and Trif*^Lps2/Lps2^* mice were orally infected and the amount of rotavirus antigen in the feces was determined at day 4 p.i.. Adult wild-type animals shed rotavirus antigen corresponding to approximately 10^6^ infectious units (IU) per dropping. In contrast, 10-fold higher levels of rotavirus antigen were excreted by adult Tlr3^−/−^ or Trif*^Lps2/Lps2^* mice (Figure 3 A). The excretion of viral antigen was additionally monitored over a time course of 9 days p.i.. Trif*^Lps2/Lps2^* mice shed rotavirus antigen from day 4 p.i. until day 8 p.i. with the highest levels around day 5 p.i., whereas wild-type animals at no time point reached comparable levels (Figure 3B). Of note, no clinical manifestation such as diarrhea was observed in adult Tlr3^−/−^ or Trif*^Lps2/Lps2^* mice and no histological damage was noted in Trif*^Lps2/Lps2^* mice, despite the elevated virus replication (Figure S1B). In contrast to the situation in adult animals, oral rotavirus infection of suckling wild-type and Trif*^Lps2/Lps2^* mice resulted in similar levels of rotavirus antigen in colon homogenates with 10^8^ IU/organ (Figure 3C and D). These results support a functionally relevant role of Tlr3 in the antiviral host response to rotavirus in adult but not neonatal IECs in accordance with a significantly enhanced expression of Tlr3 in adult *versus* neonatal IECs.

**Figure 4:**
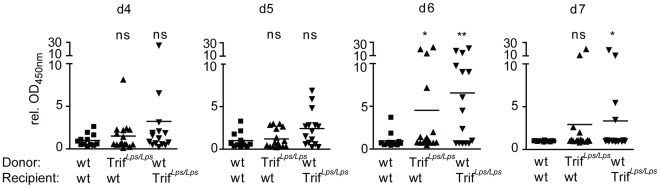
Trif-deficiency in the non-hematopoitic compartment significantly contributes to rotavirus clearance. Bone marrow chimeric wt animals reconstituted with wt bone marrow (n = 12), wt animals reconstituted with Trif*^Lps2/Lps2^* bone-marrow (n = 16), and Trif*^Lps2/Lps2^* animals reconstituted with wt bone marrow (n = 15) were generated and orally infected with rotavirus EDIM. Viral antigen shedding in feces was determined by ELISA. Values are expressed as OD_450 nm_ relative to the absorption of the wt group at 4, 5, 6 and 7 days p.i.. Data are pooled from two independent experiments. (*p<0.05; **p<0.01, Mann-Whitney test).

To verify the contribution of Trif-dependent signaling by somatic, non-hematopoietic cells such as IECs, bone-marrow chimera were investigated. Lethally irradiated wild-type mice reconstituted with wild-type bone marrow (wt→wt, n = 12), lethally irradiated wild-type mice reconstituted with bone-marrow from Trif*^Lps2/Lps2^* mice (Trif*^Lps2/Lps2^*→wt, n = 16), and lethally irradiated Trif*^Lps2/Lps2^* mice reconstituted with wild-type bone marrow (wt→Trif*^Lps2/Lps2^*, n = 15) were orally infected with rotavirus EDIM and viral antigen shedding was followed over a time course of 10 days. Whereas an only modest increase in virus shedding was observed at day 6 p.i. in chimeric Trif*^Lps2/Lps2^*→wt mice (p<0.05), a more pronounced effect was seen at day 6 and 7 p.i. in wt→Trif*^Lps2/Lps2^* animals (p<0.01 and p<0.05, respectively), consistent with a dominant contribution of Trif signaling in non-hematopoietic (e.g. epithelial) cells. Hematopoietic cells, however, also seem to contribute to the protective antiviral host response. In contrast, the wt→wt group had already cleared the infection at day 6 and 7 p.i. (Figure 4). Thus, Trif-mediated signaling in the somatic and to a lesser extent in the hematopoietic cell compartment contributes to protection from intestinal epithelial rotavirus infection.

### Characterization of the Tlr3/Trif-dependent antiviral host response

**Figure 5:**
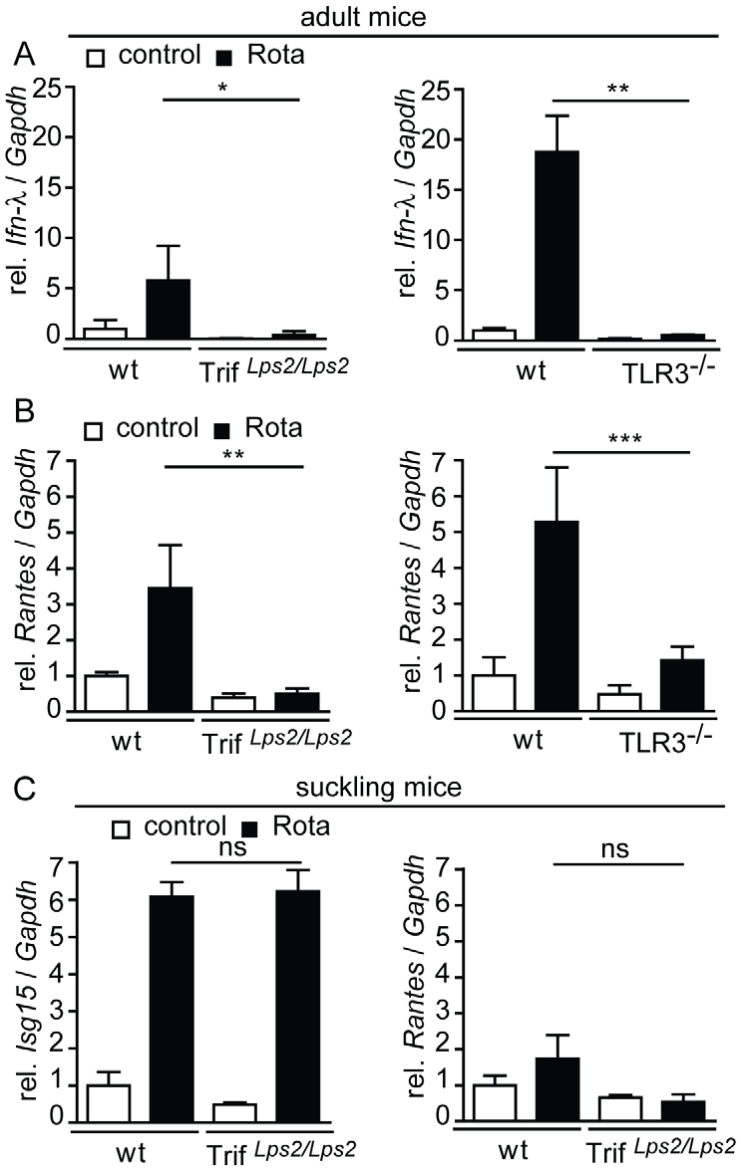
Tlr3/Trif-dependent response of adult but not neonatal IECs during rotavirus infection. (**A–C**) Quantitative RT-PCR analysis of mRNA prepared from IECs isolated from uninfected *versus* rotavirus infected adult mice at day 4 p.i.. Wt (n = 4) *versus* Trif*^Lps2/Lps2^* (n = 4, left panel) and wt (n = 4) *versus* Tlr3^−/−^ (n = 4, right panel) mice were analyzed for expression of *Ifn-λ* (**A**), and *Rantes* (**B**). Values were normalized to the expression of *Gapdh* and expressed as fold-increase over the values of the wt control group. Results are representative for at least two independent experiments. (**C**) Suckling wt (n = 3) and Trif*^Lps2/Lps2^* (n = 3) mice were orally infected with murine rotavirus EDIM. IECs were isolated at day 4 p.i. and analyzed for the expression of *Isg15* and *Rantes* (left and right panel, respectively). (*p<0.05; **p<0.01; ***p<0.001, unpaired t test).

To establish a direct link between enhanced virus shedding and diminished immune receptor-mediated stimulation of epithelial antiviral gene expression, mRNA levels of selected antiviral effector molecules were quantified in IECs prepared from wild-type, Trif*^Lps2/Lps2^*, and Tlr3^−/−^ animals (Figure 5). Consistent with a Tlr3/Trif-mediated stimulation of adult IECs after rotavirus infection, epithelial expression of *Ifn-λ* and the chemokine *Rantes* was significantly enhanced at day 4 p.i. in wild-type mice, whereas no or only weak induction was observed in IECs isolated from Trif*^Lps2/Lps2^* (Figure 5A and B, left panel) or Tlr3^−/−^ mice (Figure 5A and B, right panel). The significance of enhanced Tlr3/Trif-mediated Ifn-λ expression is illustrated by the recently reported protective effect of Ifn-λ during rotavirus infection [13].

In contrast to the situation in adult mice, expression of *Ifn-λ* and the Ifn stimulated gene *(Isg)15* was similarly induced after infection in both, wild-type and Trif*^Lps2/Lps2^* suckling mice with *Ifn-λ* mRNA levels in uninfected animals below the detection limit (Figure S2 and 5C, left panel). The chemokine *Rantes* was elevated neither in neonate wild-type nor in neonate Trif*^Lps2/Lps2^* animals (Figure 5C, right panel). These findings suggest that Tlr3/Trif-dependent epithelial signaling contributes to the antiviral response in adult mice, whereas a distinct Tlr3-independent mucosal host response is initiated in suckling mice upon rotavirus challenge.

### Recruitment of antiviral effector cells after rotavirus infection

**Figure 6:**
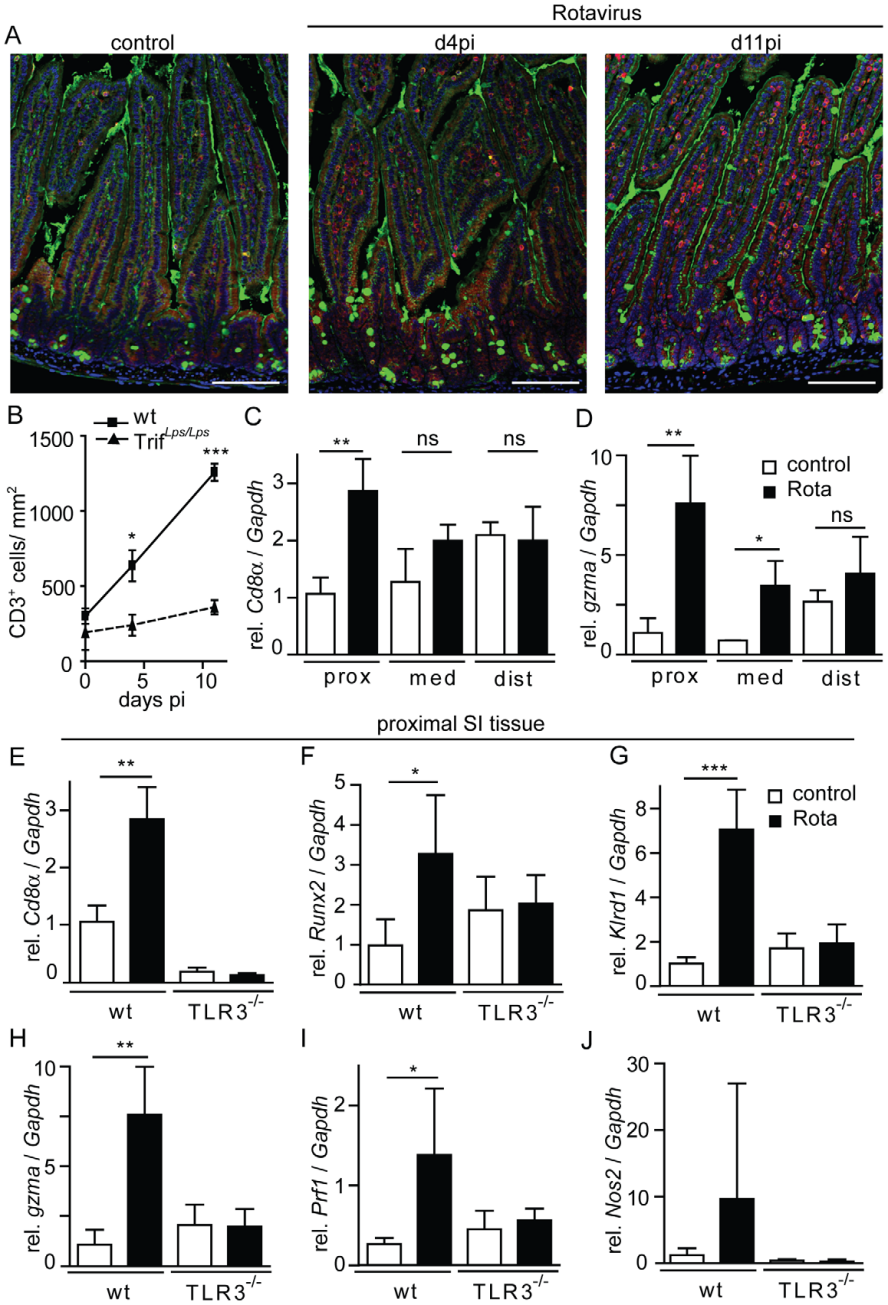
Recruitment of antiviral effector cells during rotavirus infection. (**A and B**) CD3 was stained in formalin-fixed and paraffin-embedded proximal parts of the small intestine of uninfected and rotavirus infected adult wild-type and Trif*^Lps2/Lps2^* mice at day 4 and 11 p.i. (**A**) Representative images illustrating the number of CD3^+^ cells in uninfected and rotavirus infected wild-type small intestinal tissue (CD3, red; wheat germ agglutinin, green; DAPI, blue; bar 50 µm) and (**B**) number of CD3^+^ cells quantified for 4 mice/time point and genotype. (**C and D**) Adult wt mice were orally infected with murine rotavirus EDIM and the proximal, medial and distal part of the total small intestinal tissue was analyzed at day 4 p.i. for expression levels of (**C**) *Cd8α* and (**D**) *granzyme a* (*gzma*). Uninfected wt mice served as controls. (**E–J**) The proximal part of the total small intestinal tissue of untreated or rotavirus infected wt and Tlr3^−/−^ mice was examined by quantitative RT-PCR. The values for *Cd8α* and *granzyme* expression in wt animals are identical to the data shown for proximal expression in (A) and (B). The tissue was analyzed for expression of the following marker genes of antiviral effector cells: *Cd8α* (**E**), *runt related transcription factor 2* (*Runx2*) (**F**), *killer cell lectin-like receptor subfamily D, member 1* (*klrd1*) (**G**) and expression of effector molecules *grzma* (**H**), *perforin 1* (*prf1*) (**I**), and *nitric oxide synthase 2* (*Nos2*) (**J**). Results are representative for at least two independent experiments. (*p<0.05; **p<0.01; ***p<0.001, unpaired t test).

Chemokines like Rantes facilitate recruitment of professional immune cells to the site of infection and provide antiviral host protection. To examine immune cell recruitment to intestinal tissue following rotavirus infection, immunofluorescent staining for CD3^+^ lymphocytes was performed in tissue sections of the proximal small intestine of wild-type and Trif*^Lps2/Lps2^* mice. An increase of CD3^+^ cells was observed in wild-type animals at day 4 and 11 p.i. (Figure 6A). Quantification revealed significantly higher numbers of CD3^+^ cells in intestinal tissue of infected wild-type animals compared to Trif*^Lps2/Lps2^* mice (Figure 6B). To allow better quantification of immune cell recruitment at early time points (day 4 p.i.) we performed quantitative RT-PCR for genes preferentially expressed by recruited professional immune cells using RNA from total small intestinal tissue. A marked increase of *Cd8α* and *granzyme A* (*gzma*) expression was observed in wild-type small intestinal tissue 4 days p.i. indicating recruitment of CD8^+^ effector T cells and NK cells (Figure 6C and D). Interestingly, infection-induced enhancement of *Cd8α* and *granzyme A* (*gzma*) expression was most prominent in the proximal small intestinal tissue with only minor differences observed in the distal part of the small intestine. This might reflect the relatively early time point examined during the course of infection in the context of the described proximal-to-distal progression of viral spread within the intestinal tract [14].

Subsequently, expression of *Cd8α*, *Runt-related transcription factor 2 (Runx2), killer cell lectin-like receptor D1 (klrd1), granzyme A* (*gzma*), *perforin (prf) 1* and *inducible nitrogen synthase (Nos) 2* was determined by quantitative RT-PCR in total proximal small intestinal tissue of wild-type and Tlr3^−/−^ mice 4 days p.i.. Significantly enhanced expression levels of *Cd8α* and *Runx2* in wild-type but not Tlr3^−/−^ mice indicated impaired recruitment of CD8^+^ cytotoxic T cells in the absence of Tlr3-mediated innate immune signaling (Figure 6E and F). Similarly, expression of the NK cell marker *klrd1* was significantly enhanced after rotavirus infection in wild-type, but not Tlr3^−/−^ animals (Figure 6G). Also significantly increased expression of T lymphocyte transcribed antiviral host response genes such as granzyme A and perforin was noted in rotavirus-infected wild-type but not Tlr3^−/−^ animals (Figure 6H and I). Finally, a non-significant increase of *Nos2* expression was noted in infected wild-type but not Tlr3^−/−^ animals (Figure 6J). These results indicate that antiviral effector cells, like CD8^+^ T cells and NK cells, which are critically involved in antiviral host protection, are recruited to the adult small intestinal lamina propria upon rotavirus infection in a Tlr3-dependent manner. Impaired lymphocyte recruitment in rotavirus-infected Tlr3^−/−^ adult animals might thus contribute to the enhanced virus replication observed in the absence of Tlr3-Trif-mediated cell signaling.

### TLR3 expression in human duodenal biopsies from children and adolescents

**Figure 7:**
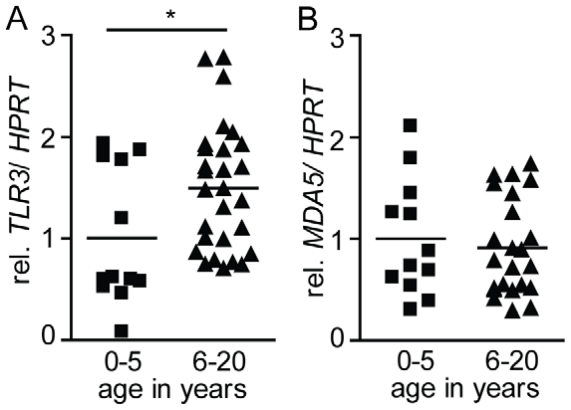
TLR3 and MDA5 expression in human small intestinal biopsies. Human endoscopic samples from the duodenum of individuals between 0 and 20 years of age were analyzed by real-time PCR for the expression level of (**A**) *TLR3* and (**B**) *MDA5* and normalized to *HPRT* (age 0–5 n = 12; age 6–20 n = 28). (*p<0.05, Mann-Whitney test).

As mentioned earlier, an age-dependent susceptibility to rotavirus is also observed in humans. Whereas rotavirus-induced gastroenteritis is frequently observed in young infants, symptomatic infection is rarely seen in individuals older than 5 years. To study a potential role of age-dependent intestinal TLR3 expression also in humans, endoscopic samples of duodenal tissue from individuals between 0 and 20 years were examined by quantitative RT-PCR. Although a large variation in the expression level was noted, the “rotavirus-resistant” group of individuals between 5–20 years of age exhibited a moderately but significantly elevated level of *TLR3* mRNA as compared to the susceptible group below 5 years of age (Figure 7A). The increase in the expression level was specific for *TLR3* as the level of *MDA5*, another innate immune receptor for viral RNA was not different between these age-groups (Figure 7B).

## Discussion

Humans and other mammals show a pronounced age-dependent susceptibility towards symptomatic rotavirus infection but the underlying mechanisms have not been defined. Intestinal epithelial cells (IEC) represent the prime target cell type for rotavirus and infection induces destruction of the intestinal epithelium with some host specific variation. A marked reduction of virus shedding and virus antigen-positive epithelial cells has been observed after infection of adult as compared to suckling mice [15], [16], although infected epithelial cells, viral replication and shedding of infectious particles are clearly also detected in adult animals [2]. An approximately 100-fold lower virus antigen shedding or viral RNA levels were observed in adult fecal samples as compared to colon homogenate from suckling mice after infection with the homotypic EDIM strain in accordance with our results [2], [17].

In addition, enhanced fluid secretion and diarrhea is observed in infected suckling but not adult animals, which may be caused by the reduced epithelial absorptive capacity, an impaired epithelial barrier function, the viral enterotoxin NSP4 causing an epithelial Ca^2+^ increase and mediating chloride secretion or a dysfunctional enteric nervous system [18]. Although rotavirus replication is restricted to the small intestine, enhanced fluid secretion is also observed in the colon. Fluid loss may therefore not be directly related to virus-induced epithelial damage and the results presented on innate host defence mechanisms that restrict viral replication may not be directly linked to the development of diarrhea. Interestingly, intestinal expression of the receptor for the neuroendocrine peptide galanin (Gal1-R), shown to be involved in rotavirus-induced fluid secretion, is restricted to the suckling period in mice [19]. Age-dependent expression of Gal1-R has therefore been proposed to contribute to the age-dependent disease manifestation of rotavirus infection.

Additional factors might, however, play a role in the enhanced susceptibility of the neonatal epithelium to rotavirus infection. In the present study, we demonstrate age-dependent expression of the viral dsRNA-recognizing innate immune receptor Tlr3 by murine IECs. Low expression levels were measured during the rotavirus susceptible postnatal period, whereas IECs isolated from adult mice exhibited significantly enhanced *Tlr3* expression. Based on the strong epithelial cell tropism of rotavirus and the established role of dsRNA in Tlr3 stimulation, we hypothesized that age-dependent differences in epithelial innate immune recognition might contribute to the protection of adult mice from symptomatic rotavirus infection [2]. The time course of increasing epithelial Tlr3 expression during the postnatal period precisely correlated with the establishment of resistance against symptomatic rotavirus infection. The low level of epithelial *Tlr3* expression failed to mediate antiviral protection after oral rotavirus infection and administration of the synthetic Tlr3 ligand poly(I∶C) induced only low transcriptional responses in neonatal IECs. Nevertheless, residual low TLR3-mediated signal transduction in neonatal IECs cannot formally be ruled out. In contrast, Tlr3/Trif-mediated signaling in adult animals induced significant upregulation of antiviral cytokines and chemoattractive mediators followed by effector cell recruitment. Rotavirus infection of adult bone marrow chimeric mice clearly demonstrated the protective role of signaling via the Tlr3 adaptor molecule Trif in non-hematopoietic cells. Thus, our data strongly suggest that an age-dependent increase in epithelial innate immune recognition *via* Tlr3 contributes to the enhanced antiviral host protection in adult animals.

Interestingly, analysis of human duodenal biopsies revealed a significantly enhanced *TLR3* expression in children of 5 years and older, correlating with the enhanced resistance to rotavirus infection in this age group. In contrast to the situation of mice bred under SPF conditions, the acquisition of adaptive immunity during childhood protects the adult human population. However, additional age-dependent innate mechanisms such as the detected age-dependent *TLR3* upregulation in mucosal tissue may also contribute to protect the adult human population. Of note, a marked individual variation of the *TLR3* mRNA level was noted and the age-dependent difference was less pronounced as compared to the situation in mice. This might in part be due to the more mature phenotype of the human intestinal mucosa at birth or the presence of TLR3-positive dendritic cells, endothelial cells and fibroblasts within the tissue biopsies [20]. Also, TLR3 was shown to be transcriptionally regulated by immune stimulation and thus the underlying disease of the patients included in the present study might have influenced the TLR3 expression, despite the fact that biopsies were obtained from apparently healthy areas of the mucosa.

Two recent papers identified B lymphocyte-induced maturation protein 1 (Blimp1 encoded by *Prdm1*) as a central regulator of the developmental gene expression program in murine IECs during the postnatal period [11], [12]. Blimp1 acts as transcriptional repressor [21] by competing with the transactivating activity of interferon regulatory factors (IRFs) [22]. Blimp1 expression is high in fetal and neonatal IECs shortly after birth but decreases during the third week of life [11], [12] precisely accompanying the postnatal increase in *Tlr3* expression described in the present study. IEC-specific Blimp1-deficient mice at birth exhibit an anatomically mature adult intestinal epithelium and show high postnatal mortality illustrating the critical role of Blimp1 for the adaptation of neonatal IECs to the conditions of the suckling period. In accordance with a regulatory role of Blimp1 for *Tlr3* expression, IRF binding sites were identified in the promoter region of both murine and human *TLR3* [23].

We have recently shown that the innate immune response to rotavirus both in the suckling and adult intestine induces a protective Ifn-λ response in the intestinal epithelium [13]. In contrast, type I Ifns failed to induce antiviral stimulation of the intestinal epithelium *in vivo* and therefore did not confer protection from rotavirus infection. A strong induction of intestinal epithelial Ifn-λ expression was noted in both, neonate and adult wild-type animals upon viral infection [13]. Importantly, enhanced *Ifn-λ* and *Isg15* expression in neonatal IECs under conditions of high viral replication was independent of Tlr3/Trif-induced signaling and presumably mediated by the RLRs Rig-I and/or Mda5. In contrast, *Ifn-λ* expression in adult animals required intact Tlr3/Trif signaling most likely in combination with RLRs.

In addition to the induced *Ifn-λ* expression, we demonstrate that also the chemokine Rantes, known to induce the recruitment of immune effector cells to the site of infection, is expressed in a Tlr3/Trif-dependent manner in adult mice. In accordance, significantly enhanced expression of lymphocyte effector proteins such as granzyme A and perforin were noted to depend on the presence of Tlr3. These results are consistent with the described association between the appearance of virus-specific CD8^+^ cytotoxic T cells and rotavirus clearance [24], [25]. Interestingly, although we show that Tlr3/Trif-dependent epithelial cell signaling significantly contributes to the recruitment of effector T lymphocytes to the proximal intestine, Trif-deficient mice similar to mice lacking the Ifn-λ receptor IL-28R successfully cleared the virus during the second week after infection [13]. Thus, impaired Tlr3/Trif-dependent signaling might enhance viral replication and prolong excretion of viral antigens. However, the resulting high viral load might in turn enforce compensatory pathways of innate immune activation via e.g. RigI or Mda5, illustrating the redundant action of innate immune recognition to ensure control of pathogenic microorganisms and efficient mucosal host protection.

The role of Tlr3 during viral infection is not uniform and both beneficial as well as detrimental effects of Tlr3-mediated immune activation have been noted [26]. A non-redundant protective role against herpes simplex virus 1 (HSV-1) encephalitis was noted in humans carrying mutations in *TLR3*, *Unc93b*, an ER protein required for TLR3, 7, 8 and 9 signaling, or *TRAF3*, a downstream adaptor protein and E3 ubiquitin ligase in TLR3 and TLR4 signaling [27]–[29]. On the other hand, a detrimental effect of Tlr3 signaling on the outcome after viral infection was noted in several animal models. For example, Tlr3-deficient mice had a reduced inflammatory response, lower virus burden and a better survival rate in a vaccinia virus infection model [30]. Also, Tlr3-deficient mice showed better survival in a murine model of influenza A virus-induced acute pneumonia, despite the impaired immune response and virus clearance [31], [32]. Finally, reduced viral titers in the periphery but penetration into the CNS and lethal encephalitis were observed after West Nile virus infection of Tlr3-deficient mice [33].

Several factors might contribute to the observed differences in the effect of Tlr3 on disease outcome after viral infection. Beside viral dsRNA recognition, Tlr3 has also been described to mediate immune activation in response to tissue disruption, a feature associated with viral cytotoxicity but also immune-mediated killing of virus-infected cells and prone to amplify inflammatory diseases [34]. Also, Tlr3 expression is highly cell type-specific and the viral spread within the tissue and the cellular composition of the inflammatory reaction might thereby influence the impact of Tlr3-mediated virus recognition. Cell type-specific differences in the cellular localization of Tlr3 with influence on the downstream signaling may also lead to variation in the antiviral response [26], [34]. Due to the lack of suitable antibodies against murine Tlr3, the cellular receptor localization in IECs has not been studied in detail. Finally, Tlr3 acts together with the RLRs and PKR in the recognition of viral dsRNA. Mda5 and RigI expression is upregulated in IECs after Tlr3 stimulation (Figure S3) and also Tlr3 itself is regulated by innate immune stimulation. The relative contribution of the RLRs, PKR and Tlr3 might therefore vary depending on the course of infection or the time point of the analysis.

Broquet *et al.* investigated in a recent study the contribution of RLRs and Tlr3 in the protective immune response to rotavirus infection. In contrast to our results, they observed no increase in viral titer in IECs in the absence of Trif after oral rotavirus infection of adult mice [8]. Instead, a critical role of Rig-I and Mda5-dependent signaling through MAVS was detected. The discrepancy observed in adult mice might be caused by the different rotavirus strain used. Whereas Broquet *et al.* used two heterotypic simian virus strains that require a higher infectious dose and exhibit lower viral replication, our results were obtained from mice infected with the homotypic highly replicating mouse EDIM strain more closely resembling natural infection. Viral immune evasion mechanisms prevent recognition in the infected cell early during the infectious cycle, whereas a large number of dsRNA molecules are produced and presumably released after virus replication during the course of infection [35]. The concentration and cellular localization of dsRNA molecules and the exposure to neighboring epithelial cells might hence account for the discrepant findings on the role of endosomal dsRNA recognition by Tlr3. Nevertheless, the helicases Rig-I and Mda5 might well contribute to the innate immune response to rotavirus in the adult intestine also in our model and explain the induction of antiviral host protection in the neonate intestine in the absence of functional Tlr3/Trif- signaling [8].

In conclusion, we describe an age-dependent upregulation of intestinal epithelial Tlr3 expression during the postnatal period and demonstrate its functional relevance in an oral rotavirus infection model. Host protection in adult IECs is shown to significantly rely on Tlr3/Trif mediated innate immune activation whereas the antiviral immune defense in suckling mice is Trif independent. Additionally, we provide evidence that intestinal TLR3 expression in humans also exhibits an age-dependent upregulation, which might contribute to rotavirus susceptibility in young infants. Our data thus illustrate the complexity of the mucosal host defence during postnatal establishment of host microbial homeostasis and identify age-dependent expression of Tlr3 as one factor of rotavirus susceptibility.

## Materials and Methods

### Ethic statement

Clinical investigations on human patient samples have been conducted according to the Declaration of Helsinki. The study was approved by the local Ethics Committee at Hannover Medical School (MHH Ethikkomission Nr. 1016-2011), and samples were obtained after informed written consent of the patient or the patient's legal representative.

All animal experiments were performed in compliance with the German animal protection law (TierSchG) and approved by the animal welfare committee of Hannover Medical School and the Niedersächsische Landesamt für Verbraucherschutz und Lebensmittelsicherheit Oldenburg (AZ 33.9-42502-04-09/1709). Animals were housed and handled in accordance with good animal practice as defined by FELASA and the national animal welfare body GV-SOLAS (www.gv-solas.de/index.html).

### Mice

Adult C57BL/6 (wt) mice were obtained from Charles River (Sulzfeld, Germany) and bred locally. C57BL/6J Ticam1 LPS2/J code 5091 (Trif*^Lps2/Lps2^*) mice [36] and TLR3^−/−^ mice [37] were bred locally. 4–6 week old mice were used for infection experiments of adult mice, whereas animals 4–8 days after birth are referred to as suckling mice. Recipients for bone marrow chimeras were lethally irradiated with a single dose of 9 Gy and transplanted with 8×10^6^ congenic bone marrow cells purified by discontinuous Lympholite-M gradient centrifugation. Chimera were infected 8 weeks after transplantation.

### Human samples

Samples were obtained from apparently healthy areas of the duodenum of children and adolescents between 0 and 20 years of age. Tissue samples were stored in Trizol, immediately stored on dry ice and RNA was purified as described below.

### Virus infections

*In vivo* infection experiments were performed, using the murine rotavirus strain EDIM provided by Lennart Svensson (Linköping, Sweden). Viral stocks for infection experiments were prepared from pooled colon content of suckling mice collected 4 days p.i.. The viral stock was quantified by using the rotavirus-antigen ELISA and serial dilution of a rhesus rotavirus stock with known infectious units (IU) resulting in a IU equivalent of approximately 2.2×10^8^ IU/mL. Suckling mice received 5 µl per os of a 1∶100 dilution of the rotavirus stock preparation, whereas adult animals (4–6 week-old) were orally infected with 40 µl of a 1∶10 dilution. Diarrhea was noted in most neonate animals at day 2 p.i. (data not shown).

### Isolation of primary intestinal cells

Epithelial cells were isolated from small intestinal tissue as recently described [38]. Briefly, the epithelial cell layer of adult mice was detached from the underlying tissue of inverted intestinal segments by incubation in 30 mM EDTA and enriched by sedimentation at 1× g. For IEC preparation from intestinal tissue of suckling mice, EDTA-treated epithelial cells were separated from the underlying tissue using a cell strainer. Flow cytometric analysis revealed less than 5% and 1% of CD45^+^ cells in IEC preparations from adult and suckling mice, respectively.

For whole tissue analysis Peyer's patches were carefully removed and the small intestine was divided into three parts (proximal, medial and distal) and shock frozen in liquid nitrogen.

For isolation of intestinal immune cells, the gut lumen was washed and Peyer's patches were removed. The intestines were opened longitudinally, washed with cold 5% FCS/PBS and incubated in RPMI 1640 with 10% FCS, 1 mg/mL collagenase D (Roche), and 1 U/mL DNase I (Sigma) for 45 min (3-day-old mice) and two times 30 min (21-day-old mice) at 37°C. After incubation the tissues were shaken vigorously for 10 s, filtered through a nylon mesh and purified by discontinuous density gradient centrifugation with 40–70% Percoll (GE Healthcare). The interphase containing the intestinal immune cells was collected and subjected to FACS analysis with about 60% and 85% of the cells being CD45 positive in suckling mice and adult mice, respectively.

### Gene expression analysis (RT-PCR and microarray)

RNA was isolated with TRIzol (Invitrogen) according to manufacturer's instruction. Reverse transcription was performed employing 1–2 µg of total RNA with RevertAid reverse transcriptase (Fermentas, St-Leon-Rot, Germany). For microarray analysis total RNA from IECs isolated from small intestinal tissue were obtained from 3-day-old and 21-day-old mice or conventional bred and germ-free-housed 28-day-old mice. Each condition was analyzed in quadruplicates. 1 µg of total RNA were used to prepare Cy3-, or Cy5-labeled cRNA using the Low RNA Input Linear Amplification Kit PLUS, Two-Color” (Agilent Technologies) according to the manufacturer's recommendations. cRNA fragmentation, hybridization and washing steps were performed exactly as recommended by the manufacturer in the “Two-Color Microarray-Based Gene Expression Analysis Protocol V5.0.1”. Slides were scanned on the Agilent Micro Array Scanner G2505 B at two different PMT settings (100% and 5%) to increase the dynamic range of the measurements (extended dynamic range mode). Data extraction and normalization were performed with the “Feature Extraction Software V9.5.3.1” by using the recommended default extraction protocol file: GE2-v5_95_Feb07.xml.

Real-time PCR was quantified using Sybr green (Invitrogen) and analyzed using the Pfaffl method to express relative expression of the target gene to the *Gapdh* housekeeping gene [39]. The following primers were used in this study: *Isg15* (forw: gagctagagcctgcagcaat, rev: ttctgggcaatctgcttctt), *Tlr3* (forw: cgaaagttggacttgtcatcaa, rev: agttgggcgttgttcaagag), *Rantes* (forw: tccaatcttgcagtcgtgtttg, rev: tctgggttggcacacacttg), *Ifn-lambda 2/3* (forw: agctgcaggccttcaaaaag, rev: tgggagtgaatgtggctcag) [40], *Cd8α* (forw: gctcagtcatcagcaactcg, rev: gtgcacaggtcagggagttc), *GrzmA* (forw. ctggcgctttgattgaaaag, rev. gattgagtgagccccaagaa), *Runx2* (forw: ccaagtagccaggttcaacg, rev. tggggaggatttgtgaagac), *Klrd1* (forw: accttctccaaccaccactg, rev: aattgcactgatgcccaac), *Pfr1* (forw: gacacagtagagtgtcgcatgt, rev: tctgagcgcctttttgaagt), *Nos2* (forw. gcttgccccaacaggagaag, rev: gctgcccggaaggtttgtac), *Mda5* (forw: agtgtcagctgcttcgatga, rev: gctcgggggatactcttttt), *RigI* (forw: ttgcaacttgctttggagaa, rev: atcatcctcatcagccttgc) and *Gapdh* (forw: tgcaccaccaactgcttagc, rev: ggcatggactgtggtcatgag) [41]. The *Tlr3* primers are predicted to detect the two reported splice variants of the murine *Tlr3* mRNA. Reverse transcription of human tissues was performed with random hexamer primers and quantitative real time PCR was done with the Applied Biosystem Taqman system. The following TaqMan Gene Expression Assays were used: TLR3: Hs01551078, MDA5: Hs01070332_m1 and HPRT: Hs01003267_m1. The expression array data are accessible through GEO Series accession numbers GSE35596 and GSE35597.

### Rotavirus-antigen ELISA

To determine the viral load in colon homogenates or stool samples, the samples were homogenized in the dilution buffer supplied with the RIDASCREEN Rotavirus Elisa Kit from R-Biopharm (Darmstadt, Germany) and the ELISA was performed according to the manufacturer's instructions. Samples were diluted to allow measurement within the linear range of the assay. For absolute quantification a serial dilution of a rhesus rotavirus stock with known infectious units (IU) was run in parallel. Of note, the detection limit of the ELISA is 10^6^ IU/mL (or 2×10^5^ IU/dropping) and therefore some of the wild-type samples with lower viral titer might be overestimated.

### Histology

Paraformaldehyde-fixed tissue sections were deparaffinized and hematoxylin and eosin staining was performed according to Mayer's protocol with reagents from Roth (Karlsruhe, Germany). For immunofluorescent staining antigen retrieval in deparaffinized paraformaldehyde-fixed tissue sections was performed with 0.01 M sodium citrate buffer. Slides were blocked with normal rat serum (Jackson ImmunoResearch, Suffolk, UK) and stained with the rabbit anti-CD3 polyclonal antiserum (Sigma) followed by a Cy3-conjugated goat-anti-rabbit secondary antibody (Jackson ImmunoResearch, Suffolk, UK). Counterstaining was performed with fluorescein-conjugated wheat germ agglutinin (WGA-Fitc, Vector Laboratories, Servion, Switzerland) and slides were mounted in DAPI-containing Vectashield (Vector Laboratories). Tissue sections were visualized using an ApoTome-equipped Axioplan 2 microscope connected to an AxioCam Mr digital Camera (Carl Zeiss MicroImaging, Inc., Göttingen, Germany).

## Supporting Information

Figure S1 **Age-dependent histological alterations after rotavirus infection (A and B).** H&E stainings of small intestinal tissue sections of uninfected (co) and rotavirus infected suckling (**A**) and adult wt and Trif*^Lps2/Lps2^* mice (**B**) at day 4 p.i. (d4 pi). Upper panel: Bar 50 µm. Lower panel: Bar 15 µm. p.i., post infection.
(TIF)

Figure S2 **Ifn-λ induction during rotavirus infection in suckling mice.** Suckling wt (n = 3) and Trif*^Lps2/Lps2^* (n = 3) mice were orally infected with murine rotavirus EDIM. IECs were isolated at day 4 p.i. and analyzed for the expression of *Ifn-λ* and normalized to *Gapdh.* Arbitary units are shown as the wt control values were below the detection limit. nd, not detectable (ns, not significant; *p<0.05; **p<0.01; unpaired t test).
(TIF)

Figure S3 **Rig-I like helicase expression during rotavirus infection.** Adult mice were orally infected with murine rotavirus EDIM and IECs were analysed at day 4 p.i. for the expression of (**A**) *Mda5* and (**B**) *Rig-I*. (*p<0.05; **p<0.01; unpaired t test).
(TIF)

## References

[1] WHO (2004).

[2] Kordasti S, Istrate C, Banasaz M, Rottenberg M, Sjovall H (2006). Rotavirus infection is not associated with small intestinal fluid secretion in the adult mouse.. J Virol.

[3] Takeuchi O, Akira S (2009). Innate immunity to virus infection.. Immunol Rev.

[4] Kawai T, Akira S (2008). Toll-like receptor and RIG-I-like receptor signaling.. Ann N Y Acad Sci.

[5] Alexopoulou L, Holt AC, Medzhitov R, Flavell RA (2001). Recognition of double-stranded RNA and activation of NF-kappaB by Toll-like receptor 3.. Nature.

[6] Sato A, Iizuka M, Nakagomi O, Suzuki M, Horie Y (2006). Rotavirus double-stranded RNA induces apoptosis and diminishes wound repair in rat intestinal epithelial cells.. J Gastroenterol Hepatol.

[7] Zhou R, Wei H, Sun R, Tian Z (2007). Recognition of double-stranded RNA by TLR3 induces severe small intestinal injury in mice.. J Immunol.

[8] Broquet AH, Hirata Y, McAllister CS, Kagnoff MF (2011). RIG-I/MDA5/MAVS are required to signal a protective IFN response in rotavirus-infected intestinal epithelium.. J Immunol.

[9] Sen A, Pruijssers AJ, Dermody TS, Garcia-Sastre A, Greenberg HB (2011). The Early Interferon Response to Rotavirus Is Regulated by PKR and Depends on MAVS/IPS-1, RIG-I, MDA-5, and IRF3.. J Virol.

[10] Vijay-Kumar M, Gentsch JR, Kaiser WJ, Borregaard N, Offermann MK (2005). Protein kinase R mediates intestinal epithelial gene remodeling in response to double-stranded RNA and live rotavirus.. J Immunol.

[11] Harper J, Mould A, Andrews RM, Bikoff EK, Robertson EJ (2011). The transcriptional repressor Blimp1/Prdm1 regulates postnatal reprogramming of intestinal enterocytes.. Proc Natl Acad Sci U S A.

[12] Muncan V, Heijmans J, Krasinski SD, Buller NV, Wildenberg ME (2011). Blimp1 regulates the transition of neonatal to adult intestinal epithelium.. Nat Commun.

[13] Pott J, Mahlakoiv T, Mordstein M, Duerr CU, Michiels T (2011). IFN-{lambda} determines the intestinal epithelial antiviral host defense.. Proc Natl Acad Sci U S A.

[14] Little LM, Shadduck JA (1982). Pathogenesis of rotavirus infection in mice.. Infect Immun.

[15] Sheridan JF, Eydelloth RS, Vonderfecht SL, Aurelian L (1983). Virus-specific immunity in neonatal and adult mouse rotavirus infection.. Infect Immun.

[16] Wolf JL, Cukor G, Blacklow NR, Dambrauskas R, Trier JS (1981). Susceptibility of mice to rotavirus infection: effects of age and administration of corticosteroids.. Infect Immun.

[17] VanCott JL, McNeal MM, Choi AH, Ward RL (2003). The role of interferons in rotavirus infections and protection.. J Interferon Cytokine Res.

[18] Lorrot M, Vasseur M (2007). How do the rotavirus NSP4 and bacterial enterotoxins lead differently to diarrhea?. Virol J.

[19] Hempson SJ, Matkowskyj K, Bansal A, Tsao E, Habib I (2010). Rotavirus infection of murine small intestine causes colonic secretion via age restricted galanin-1 receptor expression.. Gastroenterology.

[20] de Santa Barbara P, van den Brink GR, Roberts DJ (2003). Development and differentiation of the intestinal epithelium.. Cell Mol Life Sci.

[21] Saitou M, Payer B, O'Carroll D, Ohinata Y, Surani MA (2005). Blimp1 and the emergence of the germ line during development in the mouse.. Cell Cycle.

[22] Doody GM, Care MA, Burgoyne NJ, Bradford JR, Bota M (2010). An extended set of PRDM1/BLIMP1 target genes links binding motif type to dynamic repression.. Nucleic Acids Res.

[23] Heinz S, Haehnel V, Karaghiosoff M, Schwarzfischer L, Muller M (2003). Species-specific regulation of Toll-like receptor 3 genes in men and mice.. J Biol Chem.

[24] Franco MA, Greenberg HB (1995). Role of B cells and cytotoxic T lymphocytes in clearance of and immunity to rotavirus infection in mice.. J Virol.

[25] Franco MA, Tin C, Rott LS, VanCott JL, McGhee JR (1997). Evidence for CD8+ T-cell immunity to murine rotavirus in the absence of perforin, fas, and gamma interferon.. J Virol.

[26] Vercammen E, Staal J, Beyaert R (2008). Sensing of viral infection and activation of innate immunity by toll-like receptor 3.. Clin Microbiol Rev.

[27] Casrouge A, Zhang SY, Eidenschenk C, Jouanguy E, Puel A (2006). Herpes simplex virus encephalitis in human UNC-93B deficiency.. Science.

[28] Perez de Diego R, Sancho-Shimizu V, Lorenzo L, Puel A, Plancoulaine S (2010). Human TRAF3 adaptor molecule deficiency leads to impaired Toll-like receptor 3 response and susceptibility to herpes simplex encephalitis.. Immunity.

[29] Zhang SY, Jouanguy E, Ugolini S, Smahi A, Elain G (2007). TLR3 deficiency in patients with herpes simplex encephalitis.. Science.

[30] Hutchens M, Luker KE, Sottile P, Sonstein J, Lukacs NW (2008). TLR3 increases disease morbidity and mortality from vaccinia infection.. J Immunol.

[31] Le Goffic R, Balloy V, Lagranderie M, Alexopoulou L, Escriou N (2006). Detrimental contribution of the Toll-like receptor (TLR)3 to influenza A virus-induced acute pneumonia.. PLoS Pathog.

[32] Le Goffic R, Pothlichet J, Vitour D, Fujita T, Meurs E (2007). Cutting Edge: Influenza A virus activates TLR3-dependent inflammatory and RIG-I-dependent antiviral responses in human lung epithelial cells.. J Immunol.

[33] Wang T, Town T, Alexopoulou L, Anderson JF, Fikrig E (2004). Toll-like receptor 3 mediates West Nile virus entry into the brain causing lethal encephalitis.. Nat Med.

[34] Cavassani KA, Ishii M, Wen H, Schaller MA, Lincoln PM (2008). TLR3 is an endogenous sensor of tissue necrosis during acute inflammatory events.. J Exp Med.

[35] Weber F, Wagner V, Rasmussen SB, Hartmann R, Paludan SR (2006). Double-stranded RNA is produced by positive-strand RNA viruses and DNA viruses but not in detectable amounts by negative-strand RNA viruses.. J Virol.

[36] Hoebe K, Du X, Georgel P, Janssen E, Tabeta K (2003). Identification of Lps2 as a key transducer of MyD88-independent TIR signalling.. Nature.

[37] Honda K, Sakaguchi S, Nakajima C, Watanabe A, Yanai H (2003). Selective contribution of IFN-alpha/beta signaling to the maturation of dendritic cells induced by double-stranded RNA or viral infection.. Proc Natl Acad Sci U S A.

[38] Lotz M, Gutle D, Walther S, Menard S, Bogdan C (2006). Postnatal acquisition of endotoxin tolerance in intestinal epithelial cells.. J Exp Med.

[39] Pfaffl MW (2001). A new mathematical model for relative quantification in real-time RT-PCR.. Nucleic Acids Res.

[40] Sommereyns C, Paul S, Staeheli P, Michiels T (2008). IFN-lambda (IFN-lambda) is expressed in a tissue-dependent fashion and primarily acts on epithelial cells in vivo.. PLoS Pathog.

[41] Stockinger S, Reutterer B, Schaljo B, Schellack C, Brunner S (2004). IFN regulatory factor 3-dependent induction of type I IFNs by intracellular bacteria is mediated by a TLR- and Nod2-independent mechanism.. J Immunol.

