# Supplementary figures and images for: Age-Dependent TLR3 Expression of the Intestinal Epithelium Contributes to Rotavirus Susceptibility
Source: PLoS Pathog. 2012 May 3;8(5):e1002670. doi: 10.1371/journal.ppat.1002670 (PMC3343008; doi:10.1371/journal.ppat.1002670)

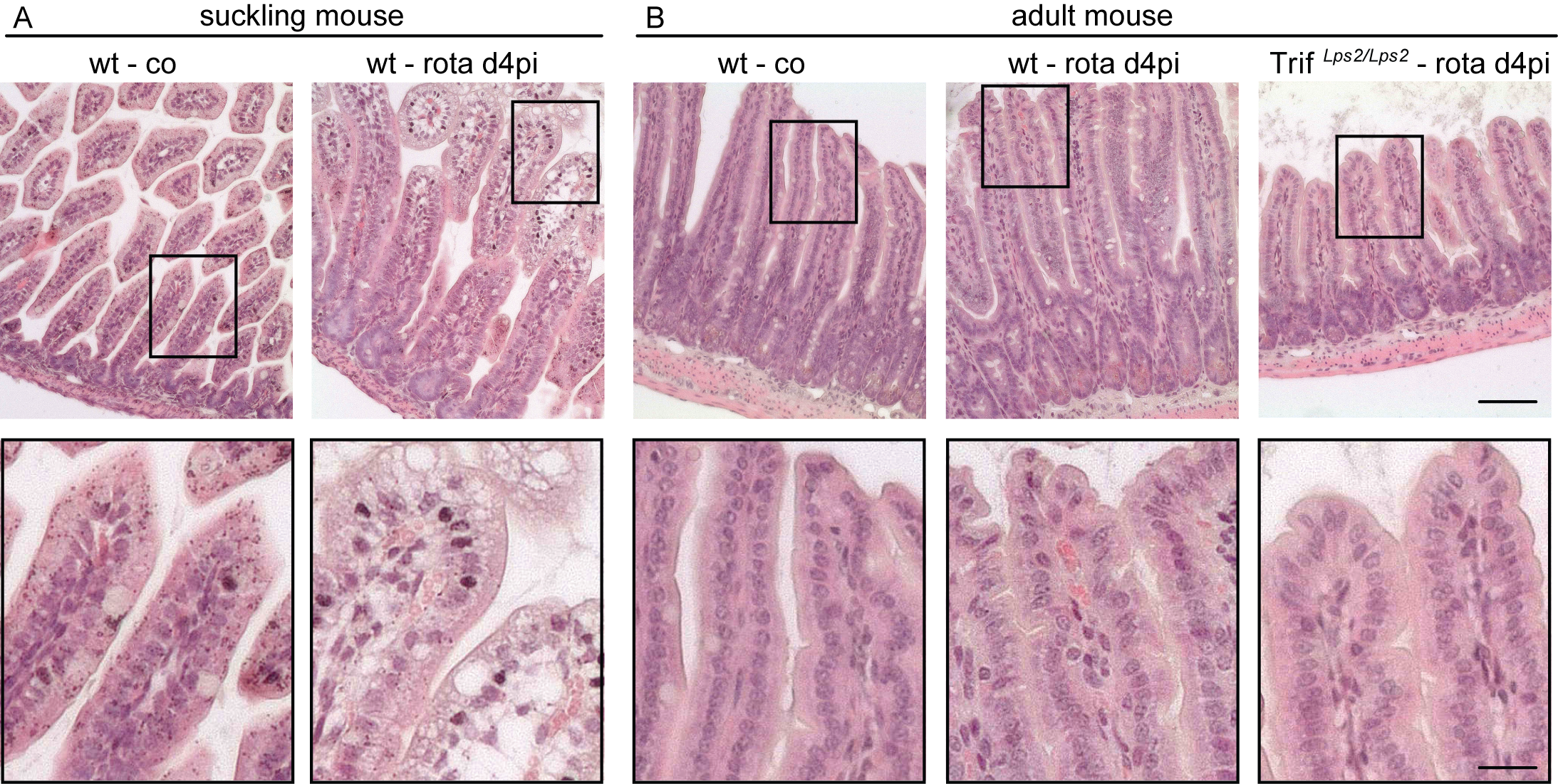

Supplement: Figure S1 — Age-dependent histological alterations after rotavirus infection (A and B). H&E stainings of small intestinal tissue sections of uninfected (co) and rotavirus infected suckling (A) and adult wt and TrifLps2/Lps2 mice (B) at day 4 p.i. (d4 pi). Upper panel: Bar 50 µm. Lower panel: Bar 15 µm. p.i., post infection. (TIF) [file ppat.1002670.s001.tif]

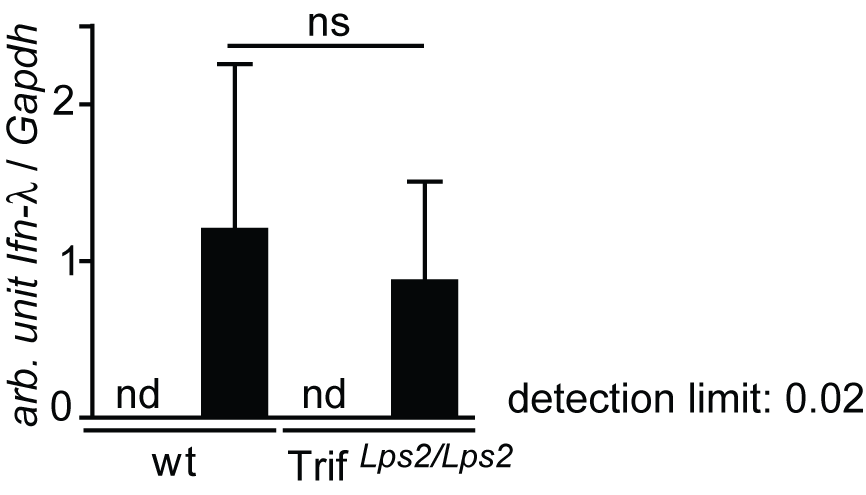

Supplement: Figure S2 — Ifn-λ induction during rotavirus infection in suckling mice. Suckling wt (n = 3) and TrifLps2/Lps2 (n = 3) mice were orally infected with murine rotavirus EDIM. IECs were isolated at day 4 p.i. and analyzed for the expression of Ifn-λ and normalized to Gapdh. Arbitary units are shown as the wt control values were below the detection limit. nd, not detectable (ns, not significant; *p<0.05; **p<0.01; unpaired t test). (TIF) [file ppat.1002670.s002.tif]

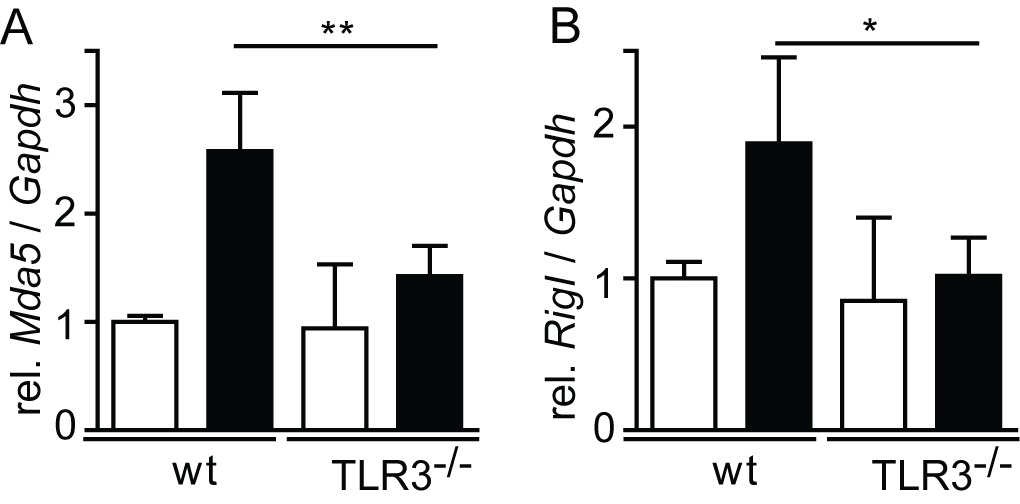

Supplement: Figure S3 — Rig-I like helicase expression during rotavirus infection. Adult mice were orally infected with murine rotavirus EDIM and IECs were analysed at day 4 p.i. for the expression of (A) Mda5 and (B) Rig-I. (*p<0.05; **p<0.01; unpaired t test). (TIF) [file ppat.1002670.s003.tif]
